# Unveiling the Secrets of the Stressed Hippocampus: Exploring Proteomic Changes and Neurobiology of Posttraumatic Stress Disorder

**DOI:** 10.3390/cells12182290

**Published:** 2023-09-15

**Authors:** Andrea Nieto-Quero, María Inmaculada Infantes-López, Emma Zambrana-Infantes, Patricia Chaves-Peña, Ana L. Gavito, Jose Munoz-Martin, Sara Tabbai, Javier Márquez, Fernando Rodríguez de Fonseca, María Inmaculada García-Fernández, Luis J. Santín, Carmen Pedraza, Margarita Pérez-Martín

**Affiliations:** 1 Departamento de Psicobiología y Metodología de las Ciencias del Comportamiento, Universidad de Málaga, 29010 Malaga, Spain; anietoquero@uma.es (A.N.-Q.); enzambrana@uma.es (E.Z.-I.); saratabbai@uma.es (S.T.); luis@uma.es (L.J.S.); 2 Instituto de Investigación Biomédica de Málaga y Plataforma en Nanomedicina-IBIMA Plataforma Bionand, 29590 Malaga, Spain; infanteslopez@uma.es (M.I.I.-L.); analugavito@hotmail.com (A.L.G.); marquez@uma.es (J.M.); fernando.rodriguez@ibima.es (F.R.d.F.); igf@uma.es (M.I.G.-F.); 3 Departamento de Biología Celular, Genética y Fisiología, Universidad de Málaga, 29010 Malaga, Spain; patricia.ch@uma.es (P.C.-P.); jmunozma@uma.es (J.M.-M.); 4 Departamento de Biología Molecular y Bioquímica, Canceromics Lab, Universidad de Málaga, 29010 Malaga, Spain; 5 Departamento de Fisiología Humana, Histología Humana, Anatomía Patológica y Educación Física y Deportiva, Universidad de Málaga, 29010 Malaga, Spain

**Keywords:** hippocampus, proteomic, posttraumatic stress disorder (PTSD), water immersion restraint stress (WIRS), natural disaster, corticosterone, hippocampal inflammation, cytokines, neuroplasticity

## Abstract

Intense stress, especially traumatic stress, can trigger disabling responses and in some cases even lead to the development of posttraumatic stress disorder (PTSD). PTSD is heterogeneous, accompanied by a range of distress symptoms and treatment-resistant disorders that may be associated with a number of other psychopathologies. PTSD is a very heterogeneous disorder with different subtypes that depend on, among other factors, the type of stressor that provokes it. However, the neurobiological mechanisms are poorly understood. The study of early stress responses may hint at the way PTSD develops and improve the understanding of the neurobiological mechanisms involved in its onset, opening the opportunity for possible preventive treatments. Proteomics is a promising strategy for characterizing these early mechanisms underlying the development of PTSD. The aim of the work was to understand how exposure to acute and intense stress using water immersion restraint stress (WIRS), which could be reminiscent of natural disaster, may induce several PTSD-associated symptoms and changes in the hippocampal proteomic profile. The results showed that exposure to WIRS induced behavioural symptoms and corticosterone levels reminiscent of PTSD. Moreover, the expression profiles of hippocampal proteins at 1 h and 24 h after stress were deregulated in favour of increased inflammation and reduced neuroplasticity, which was validated by histological studies and cytokine determination. Taken together, these results suggest that neuroplastic and inflammatory dysregulation may be a therapeutic target for the treatment of post-traumatic stress disorders.

## 1. Introduction

Postraumatic stress disorder (PTSD) is a disabling and heterogeneous psychiatric disorder, accompanied by a range of distress and treatment-resistant symptoms and that can appear after exposure to direct or indirect exposure to a traumatic event, with an emphasis on the extraordinary magnitude of the event traumatic events [1]. It that may be associated with a number of other psychopathologies, including depression and anxiety disorders and substance abuse [2,3,4]. The cause of this high heterogeneity in which numerous subtypes have been described depends on, among other factors, the type of stressor that provokes the disorder, particularly human-made trauma and natural disasters [5]. Clinically, although the prevalence of PTSD is approximately 3–9% of the population [6,7], the increase in societal challenges that has occurred recently, such as the COVID-19 pandemic, the post-pandemic period, military conflicts, and the global refugee crisis, among many other situations that expose people to intense stressful experiences, may lead to PTSD. Due to climate change, there is an increased risk of flooding, which is one of the biggest natural disasters worldwide, and the prevalence of PTSD following this natural disaster is quite high [8]. Although PTSD is a major health problem, its therapeutic approach is still limited, and mainly based on psychological therapy, which is effective in about 50% of cases [9]. Identification of the neurobiological changes in stress-induced pathology is critical for devising a means of mitigating the severity of traumatic disorders.

From a neurobiological point of view, the hippocampus is one of the key brain structures in mediating PTSD symptomatology [10]. In fact, the hippocampus, a key brain region controlling cognition and emotion, is among the most highly sensitive regions of the brain strongly influenced by stress and a target structure for the adverse effects of stress [11,12]. Moreover, this region plays a central role in regulating stress hormones and responses through the hypothalamic–pituitary–adrenal (HPA) axis, and in turn, it is also susceptible to the toxic effects of elevated glucocorticoids [13]. Posttraumatic stress disorder (PTSD) is associated with abnormal hippocampal function; however, the neurobiological changes taking place in the hippocampus that may explain, at least in part, the symptoms associated with PTSD which are poorly understood and warrants further attention. If the neurobiological changes responsible for the development of PTSD are poorly understood in general, much less is known about the changes induced by specific stressors such as natural disasters.

Proteomics is a promising strategy for characterizing the neurobiological mechanisms underlying the development of PTSD [14]. In fact, proteins are essential for cellular adaptation to environmental signals and cellular aspects of disease development [15,16]. In addition, proteomics includes levels of analysis that provide insight into posttranslational modifications and understanding the protein–protein interaction complexes and information-processing capabilities of signal transduction networks. This in turn provides insight into how those networks are disrupted in disease and may illuminate the amazing breadth and complexity of the signal transduction pathways that cells employ to respond to stress. Biological networks associated with short-term responses to trauma exposure may also predict longer-term risk for medical comorbidities [17].

Since intense stress, especially traumatic stress, can trigger disabling responses and in some cases even lead to the development of PTSD [9], understanding the neurobiological changes that occur after exposure to intense stress and can cause maladaptive responses to stress is crucial. While most efforts have focused on studying the neurobiological mechanisms that take place in the long term, many changes can be initiated following an acute stressor, particularly if it is intense, which can provide valuable insights into the mechanisms involved in the stress response and pathological responses to stress.

The main tool to gain insight into the neurobiological mechanisms associated with PTSD is animal models. These models can unravel the cellular and molecular mechanisms associated with PTSD, which can be the starting point for developing preventive strategies and designing new therapeutic targets for a disorder [9].

For these reasons, the aim of this study was, on the one hand, to determine whether an acute and intense stressor combining water immersion and movement restriction (WIRS) can induce PTSD-associated symptoms and could be used as an animal model of PTSD induced by exposure to flooding. On the other hand, we aimed to characterise the molecular profile of the initial changes that take place after exposure to this stressor. However, PTSD requires a multidimensional approach that examines multiple biological levels (molecular, cellular, etc.), which is why we subsequently corroborated the profile of hippocampal changes determined by proteomics through immunocytochemistry and molecular studies. The results revealed that this type of stress induced anhedonia, which was maintained one week later, signs of anxiety and discomfort, and altered the HPA in a manner reminiscent of that observed in PTSD. Moreover, in the hippocampus, it caused changes in the protein profile, indicating increased inflammation and reduced plasticity, as confirmed by cytochemical studies. A better understanding of the neurobiological mechanisms that take place in the initial stages of PTSD onset may increase the opportunity to implement effective preventive strategies and avoid the development of the pathological response following exposure to traumatic events [18].

## 2. Materials and Methods

### 2.1. Animals and General Procedure

Seventy-seven male C57BL/6J mice aged 3 months (Charles River Laboratories, Inc., Wilmington, MA, USA) were individually housed under standard conditions: temperature, 22 ± 2 °C; relative humidity, 55 ± 5%; 12 h light/dark cycle, lights on at 7:30 a.m.; and water and food *ad libitum*. The experiments were carried out between 9:00 a.m. and 3:00 p.m.

**Figure 1:**
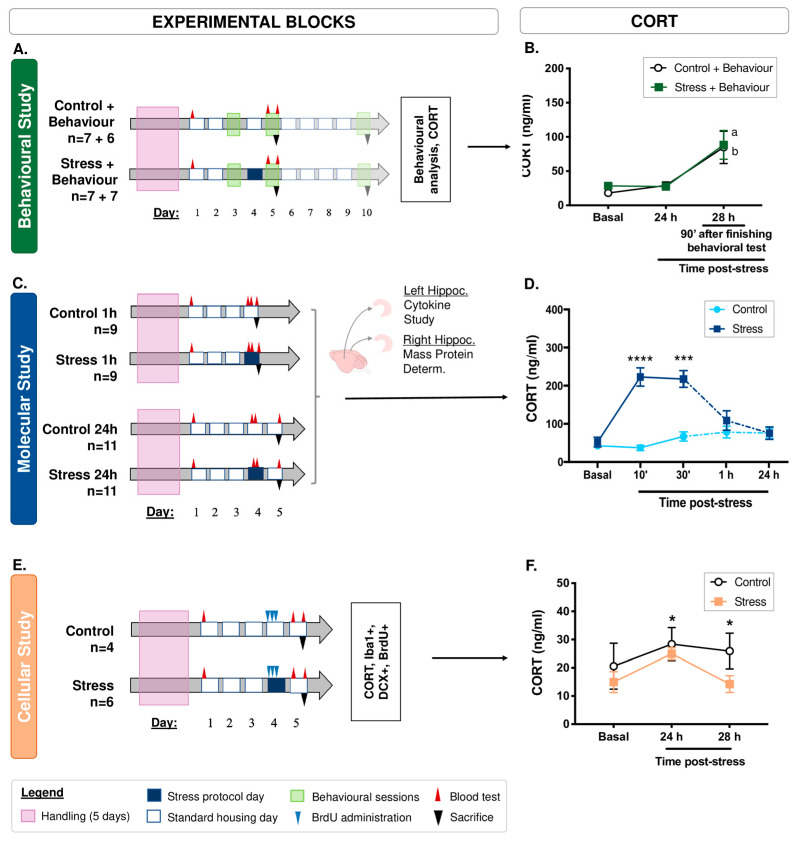
Experimental blocks with an acute WIRS protocol and evolution of CORT levels. (**A**) First experimental block to analyse the evolution of animal behaviour with a WIRS protocol. On Day 5 of the experiment, a subset of the control (n = 7) and stressed (n = 7) animals were sacrificed. The remaining control and stressed animals were sacrificed on Day 10. (**B**) CORT levels. No significant differences were observed 24 h after the application of an acute stressor, but significant differences were observed 90 min after finishing the behavioural tests in both behavioural groups, indicating a possible stressor effect of these tests. ‘a’ and ‘b’ indicate significant differences (*p* ≤ 0.05) in ‘Control + Behaviour’ or ‘Stress + Behaviour’ animals compared to the previous assessment without behavioural testing using repeated-measures ANOVA. (**C**) Second experimental block for molecular studies. The left hippocampus (Hippoc.) of each animal was used for the cytokine study and the right hippocampus was used for mass protein determination and validation by Western blotting. (**D**) CORT levels increased 10 and 30 min after stress application (Student’s *t* tests). The continuous lines represent the data for all groups, since the treatment was the same up to that time. The dashed lines refer to the means obtained at 1 h (Control and Stress 1 h) and 24 h (Control and Stress 24 h) after the application of the stressor for each group. (**E**) Third experimental block to study the effects of acute stress on neurogenesis and the hippocampal microglial response. (**F**) Significant differences in CORT levels were observed 24 and 28 h after the application of an acute stressor (environmental treatment factor from repeated-measures ANOVA). * *p* ≤ 0.05; *** *p* ≤ 0.005; **** *p* ≤ 0.0005 degrees of significance between two measures (Control vs. Stress).

To investigate the effects of acute stress at the behavioural, molecular, and cellular levels, three experimental blocks were designed. (I) Animals were subjected to acute stress and behavioural testing (Control + Behaviour, n = 13; Stress + Behaviour, n = 14) (Figure 1A). On Day 5 of the experiment, control (n = 7) and stressed (n = 7) animals were sacrificed, and the remaining animals (control, n = 6 and stressed, n = 7) were kept for 6 days post-stress to study the evolution of the preference for saccharine. (II) The animals were subjected to acute stress to study molecular parameters at 1 h (Control 1 h, n = 9; Stress 1 h, n = 9) and at 24 h post-stress (Control 24 h, n = 11; Stress 24 h, n = 11) (Figure 1C). (III) The animals were subjected to acute stress to study cellular parameters (Control, n = 4; Stress, n = 6) (Figure 1E). An overview of the studies in each experimental block can be found in Table 1.

The procedures were approved by the Ethics Committee of University of Malaga (CEUMA 2-2019-A; date: 3 February 2019) and Junta of Andalucía (08-7-15-273; date: 7 August 2015) and carried out in compliance with European animal research laws (European Parliament and Council Directives 2010/63/UE, 90/219/CEE, Regulation (EC) No. 1946/2003) and Spanish National and Regional Guidelines for Animal Experimentation (Real Decreto 53/2013, Ley 32/2007 and Ley 9/2003).

### 2.2. Stress Procedure

As a stress procedure, an acute and intense stress protocol has been applied that may be reminiscent of PTSD-inducing stress [5,9,19]. For this purpose, animals assigned to a stress group were subjected to an acute stress protocol by water immersion restraint stress (WIRS) [20,21]. For this procedure, animals were restrained in a 50 mL tube with some holes and immersed in a water bath at 22 ± 1 °C to the level of the sternum for 2 h. The control animals were kept in standard housing conditions.

### 2.3. Corticosterone Measurement

Because corticosterone (CORT) is one of the principal mediators of the impact of stress on the brain and behaviour, the blood levels of this hormone were measured [22,23,24]. At least 3 corticosterone (CORT) measurements were taken in each animal. Basal and post-stress blood samples from the lateral tail vein were collected in EDTA tubes at 9:00 am and 3:00 pm. For details of the timing of blood collection in each experimental block and process, see Figure 1 and the Supplementary Information.

### 2.4. Behavioural Testing

The behavioural tests were carried out in the control and stressed groups and performed according to our previous studies [22,23,24,25].

#### 2.4.1. Open Field Test (OFT)

The OFT was selected to evaluate the basal locomotor and exploratory activity of the animals at the beginning of the experiment. The apparatus consisted of a plastic box with dimensions of 40 × 40 × 30 cm (length × width × height). Each mouse was placed into the centre of the box and allowed to explore it for 5 min. Ethological (wall rearing, rearing, and grooming) and spatiotemporal parameters were analysed. The time spent and distance travelled in the centre and peripheral zones were analysed by Ethovision XT software (Ethovision version 12, Noldus, The Netherlands).

#### 2.4.2. Saccharin Preference Test (SPT)

Anhedonia, which can broadly be defined as a diminished capacity to experience pleasure, is a core symptom in mood disorders [22,23,25,26,27]. For this reason, hedonic behaviour was assessed after the animals were individualized and accustomed to the presence of two bottles for three days. Two measurements were taken: 24 h before stress (baseline) and 24 h after stress. A third group was assessed 6 days after the application of the stressor. The consumption of water and 0.05% saccharin solution (saccharin sodium salt hydrate; Sigma–Aldrich, Madrid, Spain) was evaluated in the control and stress groups. Saccharin preference was calculated according to the following formula:

(1) $$Saccharin\;Preference\;\left(\%\right)=100\;\cdot \;\frac{saccharin\;intake}{saccharin\;intake+water\;intake},$$

Values below 80% for saccharin preference were considered indicative of anhedonia [26,27].

#### 2.4.3. Elevated Plus Maze (EPM)

Since PTSD shares neurobiological features with anxiety disorders and may appear as one of the symptoms of this disorder, the anxiety of the animals subjected to WIRS was assessed [28,29,30,31]. The EPM was used to assess the anxiety of the animals 24 h after acute stress exposure. The EPM consisted of two open arms (30 cm long × 5 cm wide; 90–100 lux) and two enclosed arms (30 cm long × 5 cm wide × 1 cm high walls; 25–30 lux) connected to a common central platform (5 cm × 5 cm) and was elevated 38.5 cm above the floor. Ethological (wall rearing, rearing, head dipping and grooming) and spatiotemporal parameters were analysed. The time spent and distance travelled in each zone and the time/frequency ratio in open arms were analysed by Ethovision XT software (Ethovision version 12, Noldus, The Netherlands). Moreover, an anxiety index (*A.I.*) was calculated using the following formula:

(2) $$A.I.=1-\left(\frac{\frac{open\;arms\;time\;\left(s\right)}{test\;duration\;\left(s\right)}+\frac{number\;of\;open\;arms\;entries}{total\;number\;of\;entries}}{2}\right),\;$$

#### 2.4.4. Tail Suspension Test (TST)

To assess the animals’ coping with an aversive and inescapable situation, an automated TST was performed 1 h after finishing the EPM. The animals were suspended by their tails with adapted adhesive tape and attached to a hook that was coupled to a computer-assisted device for measuring movement (Panlab, Barcelona, Spain). Each testing session lasted 6 min, during which the performance of each mouse was evaluated. Immobility, energy, and power of movement (PM) were registered using a computerized system connected to the apparatus.

### 2.5. Hippocampal Molecular Analysis

A second experimental block was proposed to study the effect of acute stress on molecular parameters 1 h and 24 h post-stress (Figure 1C). The animals were anaesthetized with sodium pentobarbital (200 mg/kg) and intracardially perfused with 0.1 M phosphate-buffered saline, pH 7.4 (PBS). The brains were collected and stored at −80 °C until processed.

#### 2.5.1. Cytokine Measurement

Dysregulation of cytokines is associated with post-traumatic stress disorder (PTSD) [32]. The proinflammatory cytokines IFN-γ, IL-6, and TNF (Tumor Necrosis Factor)-α were assessed. The left hippocampi were homogenized in lysis buffer, and after centrifugation (14,000× *g*, 15 min, 4 °C), each supernatant was recovered and brought to a protein concentration of 7 μg/μL. Next, the ProcartaPlex™ Multiplex Immunoassay (Invitrogen, Thermo Fisher Scientific, Waltham, MA, USA) was performed according to the manufacturer’s directions. The detection of these cytokines was carried out on Luminex™ equipment (Bio-Plex™ 200 System, Bio-Rad, Hercules, CA, USA).

#### 2.5.2. Mass Protein Determination: Hippocampal Protein Profile

Quadrupole-Orbitrap mass spectrometry (Q-Orbitrap-MS) was used for the massive identification and quantification of hippocampal proteins. This technique separates the molecules based on their mass/charge using the shotgun proteomics approach.

For each condition (1 h and 24 h post-acute stress), a functional enrichment study was performed. The significantly overexpressed and underexpressed proteins of the right hippocampi were filtered using the following parameters: *p* < 0.05; false discovery rate (FDR) < 0.05; and fold change (FC) > 1.2 for proteins overexpressed under stress and FC < 0.833 for proteins underexpressed under stress [33]. Through STRING, using the 1st Shell option, the number of interactors was increased to 100 for both over- and underexpressed proteins. Then, MCODE was used as a clustering tool for the protein–protein interaction network obtained from STRING. The data extracted from MCODE can be found in Supplementary Information. Next, a functional enrichment analysis was performed using DAVID [34,35] by introducing each protein gene name grouped into the clusters from the previous step. The biological processes in each of the different signalling pathways (protein clustering) were determined, which in turn determined what functions were affected. Finally, a network analysis of the GO terms obtained from DAVID was performed so that it was easier to interpret and integrate the biological implications of the results.

#### 2.5.3. Western Blotting

Western blotting was performed on a selection of relevant proteins that were over- or underexpressed following Orbitrap to validate the results of the functional enrichment. For a more detailed description, please see the Supplementary Information.

### 2.6. Histology

#### 2.6.1. Immunolabelling

Mice were anaesthetized with 200 mg/mL sodium pentobarbital and intracardially perfused with PBS, pH 7.4, and 4% paraformaldehyde. The brains were post-fixed and cut into six series of coronal sections (40 µm) using a vibratome (Leica VT1000S, Leica Biosystems). The following primary antibodies were used to identify microglia and immature neurons: rabbit anti-Iba1 (1:500; Wako, ref: 019-19741) and goat anti-DCX (1:200; Santa Cruz Biotechnology, ref: sc-8066). Then, we used the corresponding biotinylated secondary antibodies: anti-rabbit (1:1000; Dako, ref: E0432) and anti-goat (1:1000; Dako, ref: E0466) [36].

To identify new neurons, control and stress mice received three intraperitoneal administrations of BrdU (50 mg/kg, Sigma–Aldrich, Madrid, Spain) separated by 3 h just after acute stress treatment. Double immunofluorescence labelling was carried out combining the rat anti-BrdU antibody (1:1000; Accurate Chemical, ref: OBT0030) and the rabbit anti-DCX antibody (1:600; Abcam, ref: ab18723), followed by the corresponding secondary antibodies of Alexa Fluor 488 anti-rat (1:1000; Invitrogen, ref: A21208) and Alexa Fluor 568 anti-rabbit (1:1000; Invitrogen, ref: A10042). DAPI was used as a nuclear contrast stain [36,37] (see Supplementary Information for details).

#### 2.6.2. Morphological Analysis of Iba1+ and DCX+ Cells and Cell Count

Iba1+ cells. The morphometric parameters of the cell soma (area, perimeter, circularity, and roundness) and distribution (density, distance and regularity index) were determined [38]. For a more detailed description of the soma study procedure, please see the Supplementary Information.

Additionally, morphological activation phenotype clustering was carried out. For this purpose, hierarchical clustering was employed using SPSS (IBM SPSS Statistics for Windows, version 25.0. IBM Corp. Armonk, New York, NY, USA). The strategy used was maximum distance or minimum similarity. Microglia located in the dentate gyrus of the hippocampus in adult male mice were considered homeostatic or vigilant microglia (under physiological situations), and microglia that changed their state due to the application of stressors were considered stress-reactive microglia [39].

DCX+ cells. DCX+ cells were classified into three degrees of maturity based on morphology: type A, proliferative or more immature cells without prolongations; type B, immature neurons with one prolongation; and type C, neurons with at least one branch in some of their prolongations extending into the molecular layer [40]. New neurons were determined by labelling colocalization for BrdU and DCX.

The numbers of Iba1+, DCX+, and BrdU/DCX+ cells were calculated for each animal (see Supplementary Information).

### 2.7. Statistical Analysis

All results are presented as the mean ± SEM. Prior to data analysis, Levene’s test was performed to determine the normality and homoscedasticity of the data and thus corroborate the relevance of the use of parametric techniques. The control and stress groups were compared by Student’s *t* test. For repeated measures (SPT), a repeated-measures ANOVA was conducted followed by a post hoc Fisher’s least significant difference (LSD) analysis when applicable. Data were considered statistically significant at *p* ≤ 0.05.

#### Principal Components Analysis (PCA)

To determine the relationship between behavioural variables and to reduce them to a smaller group of factors that would underlie stress effects, a PCA with varimax rotation was subsequently performed. Only a subset of the behavioural parameters was selected so that the analysed samples from the groups would meet statistical adequacy criteria [41]. The correlation matrix of the whole sample of animals (n = 28 control and stressed animals) was used for the analysis and tested for sampling adequacy by the Bartlett sphericity and the Kaiser–Meyer–Olking (KMO) tests. The resulting factors with eigenvalues > 1 were selected. ‘Factor loading’ (i.e., the contribution of each variable to a factor) was considered significant when it was >0.50. Finally, considering that the component or factor scores represent the relative contribution or weight of each loading pattern for each case, Student’s *t* test was used to determine whether differences existed between the groups in a given loading pattern.

## 3. Results

### 3.1. CORT Analyses

CORT determinations were carried out in each of the three experimental blocks.

Twenty-four hours after the end of the stress protocol, no differences from baseline were observed in CORT levels. However, repeated-measures ANOVA revealed a significant increase in corticosterone levels 90 min after finishing behavioural procedures in both groups (F(2, 26) = 14.30, *p* < 0.0005, LSD: *p* < 0.005) (Figure 1B), possibly due to an effect of the behavioural tests.

For the molecular study, measurements were taken at baseline and 10 min, 30 min, 1 h and 24 h post-stress. A significant increase in CORT levels was observed at 10 (t(33) = −6.51, *p* < 0.0005) and 30 min post-stress ((t(32) = −3.87, *p* < 0.005), with all other measurements being similar to baseline (t(13) = −1.05, *p* > 0.05; t(19) = −0.003, *p* > 0.05; 1 h and 24 h, respectively) (Figure 1D).

In the histological study, repeated-measures ANOVA revealed statistically significant results for the environmental treatment factor (F(1, 4) = 11.83, *p* < 0.05). As expected, LSD revealed no baseline differences between groups. However, 24 and 28 h after finishing the stress procedure, experimental animals showed significantly lower levels of CORT than control animals (LSD: *p* < 0.05) (Figure 1F).

In summary, the results of the t-student and repeated measures ANOVA for analyses of CORT levels after behavioural testing, molecular and cellular studies are shown in Table 2.

### 3.2. Basal Locomotor and Exploratory Activity Measured by OFT

**Figure 2:**
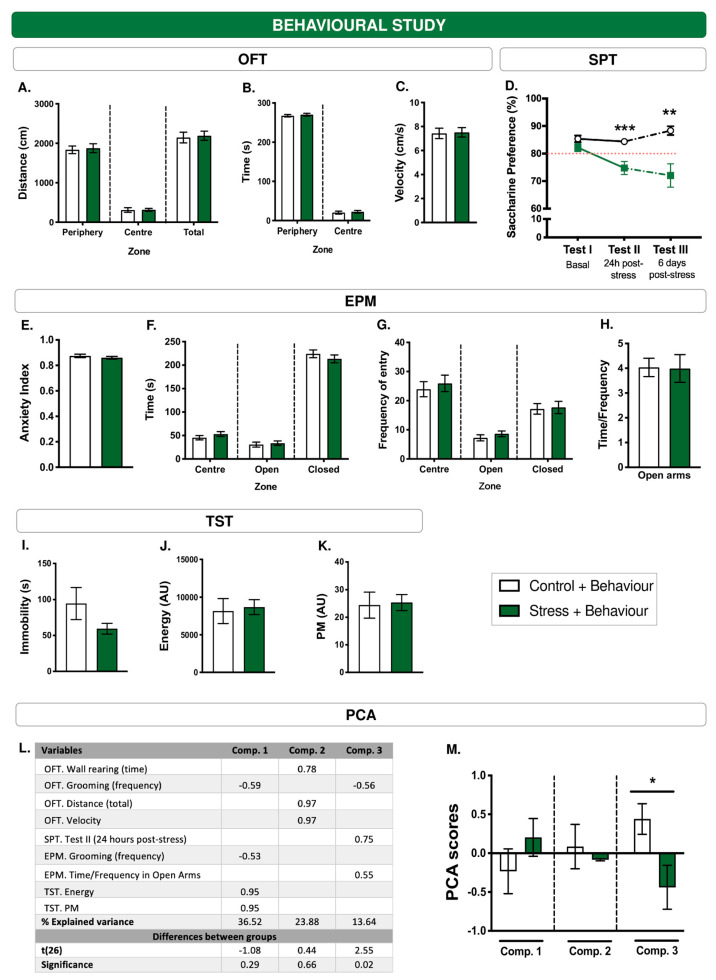
Acute WIRS-type stress exposure has an anhedonic effect at the behavioural level. Along with stress, a battery of behavioural tests modelling depression and anxiety were applied. (**A**–**C**) Basal levels of animals’ locomotor activity in the OFT. (**D**) Preference for saccharin assessed by SPT at baseline (Test I), 24 h post-stress (Test II) and 6 days post-stress (Test III). The red discontinuous line indicates the minimum threshold of preference for the 0.05% saccharin solution. Anxiety levels (**E**), locomotor activity (**F**,**G**), and time/frequency ratio on open arms (**H**) of the animals in the EPM. (**I**–**K**) Immobility, energy and power of movement measures in the TST. (**L**) Data from the PCA performed with relevant behavioural outcomes. Negative scores indicate an inverse correlation to the component (Comp.). KMO = 0.57; χ^2^ = 243.20; *p* = 0. In addition, Student’s *t*-statistics for PCA scores in each component are provided. (**M**) PCA scores for each component and group (‘Control + Behaviour’ and ‘Stress + Behaviour’). * *p* ≤ 0.05; ** *p* ≤ 0.01; *** *p* ≤ 0.005 differences between groups using Student’s *t* test.

Before environmental treatment, the animals assigned to the stress and control groups did not show significant differences in spatiotemporal (Figure 2A–C) and ethological parameters (for a detailed description of the results, please see the Supplementary Information and Figure S1A,B).

### 3.3. WIRS Causes Anhedonic Behavior

The repeated-measures ANOVA was statistically significant (F(1, 26) = 14.54, *p* < 0.005). There were no significant differences between the control and stress groups in saccharin preference at baseline (LSD: *p* > 0.05). However, after 24 h, stress caused a significant reduction in saccharin preference (LSD: *p* < 0.0005). The repeated-measures ANOVA performed on the additional groups showed that anhedonic behaviour was maintained 6 days after the end of the stressor (F(2, 22) = 5.94, *p* > 0.01; LSD: *p* < 0.005) (Figure 2D).

### 3.4. WIRS Did Not Affect the Parameters Assessed by the EPM

The EPM was used to assess anxiety-like responses in animals after the application of the acute stressor. None of the parameters examined were significant (Figure 2E–H). For a more detailed description, please see the Supplementary Information and Figure S1C,D.

### 3.5. Application of WIRS-Type Acute Stress Does Not Have Negative Consequences on Passive Stress-Coping Behaviour

The TST was administered the day after the application of acute stress and 1 h after the EPM. Stress did not increase immobility (t(26) = 1.58, *p* > 0.05; Figure 2I) or affect the energy parameter (t(26) = −0.28, *p* > 0.05; Figure 2J), power of movement, or PM (t(26) = −0.17, *p* > 0.05; Figure 2K).

In summary, the results of the t-student and repeated measures ANOVA for behavioural analyses are shown in Table 3.

### 3.6. WIRS Increased Anhedonic Behaviour and Some Parameters Related to Discomfort and Anxiety

A principal component analysis (PCA) was conducted to study the relationship among behavioural changes induced by treatments. PCA with variance-maximizing (varimax) rotation revealed a three-component solution accounting for 71.25% of the total variance. Component 1 had a negative correlation with the frequency of grooming in the OFT (−0.59) and EPM (−0.53), and a positive correlation with PM (0.95) and energy (0.95) in the TST. Component 2 had a positive correlation with wall rearing in the OFT (0.78), distance total in the OFT (0.97), and velocity (0.97). Component 3 had a negative correlation with the frequency of grooming in the OFT (−0.56) and a positive correlation with saccharin preference 24 h post-stress (0.75) and the time/frequency ratio in the open arms in the EPM (0.55). Component 1 was categorized as ‘discouraged behaviour’, Component 2 as ‘locomotion’, and Component 3 as ‘resilience to PTSD-associated symptoms’. Stressed animals scored higher on Component 1 and lower on Component 2, although only Component 3 reached statistical significance (t(26) = 2.54, *p* ≤ 0.05), showing that stressed animals had lower scores on the resilience to the development of PTSD component after stress (Figure 2L,M).

### 3.7. Among the Cytokines Studied, IL-6 Appears to Be the Most Sensitive to the Effects of ‘Stress’

**Figure 3:**
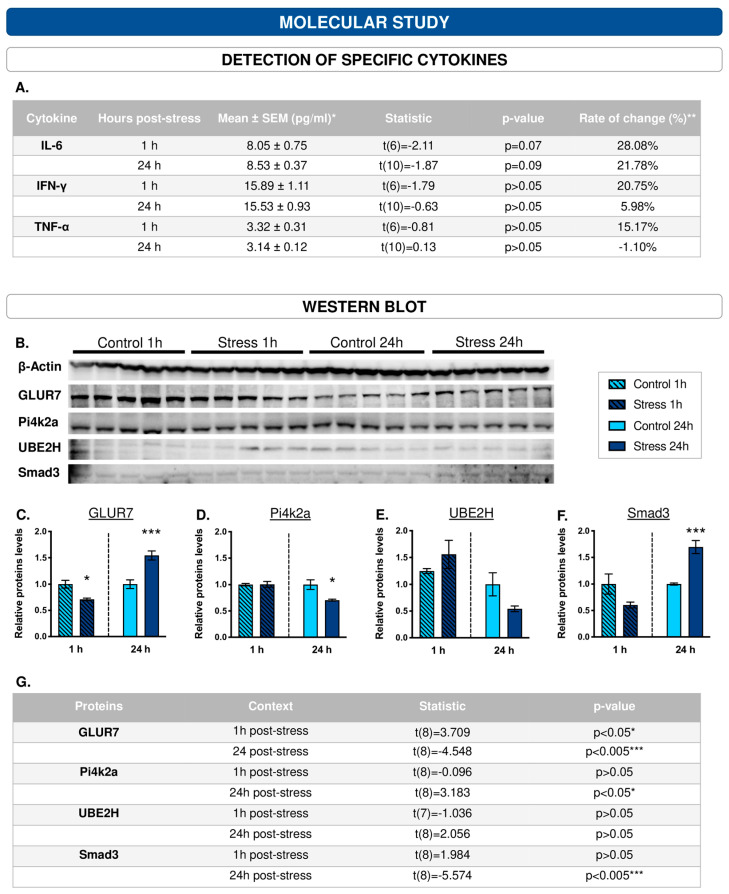
Western blots validate the effects of acute stress on the hippocampal protein profile measured by Q-Orbitrap-MS. (**A**) Cytokines studied with Luminex technology. (**B**) Representative micrograph of Western blot (consult Figure S2 for complete membranes) of proteins whose abundance (as measured by Q-Orbitrap-MS) was modified 1 h and 24 h post-acute stress (n = 5, for each group). Western blot analysis demonstrated low levels of GLUR7 in the hippocampus at 1 h following acute WIRS-type stress exposure (**C**) and Pi4k2a at 24 h (**D**). Furthermore, Western blot analysis demonstrated high levels of UBE2H in the hippocampus at 1 h following acute WIRS-type stress exposure (**E**) and Smad3 at 24 h (**F**); * *p* ≤ 0.05, ** *p* ≤ 0.01; *** *p* ≤ 0.005 differences for the stress group compared to their respective controls. (**G**) Statistical results of protein determinations by Western blot * *p* ≤ 0.05, *** *p* ≤ 0.005 differences for the stress group compared to their respective controls.

Stress increased the levels of the three proinflammatory molecules, although the differences were not statistically significant (for statistical results, please see Figure 3A).

Among them, the most sensitive was IL-6. Compared to the control group, IL-6 levels were 28.08% (t(6) = −2.11, *p* = 0.07) and 21.78% (t(10) = −1.87, *p* = 0.09) higher in the experimental group at 1 and 24 h post-stress, respectively.

### 3.8. The Protein Profile of the Hippocampus Varies after 1 h and 24 h of WIRS Exposure

**Figure 4:**
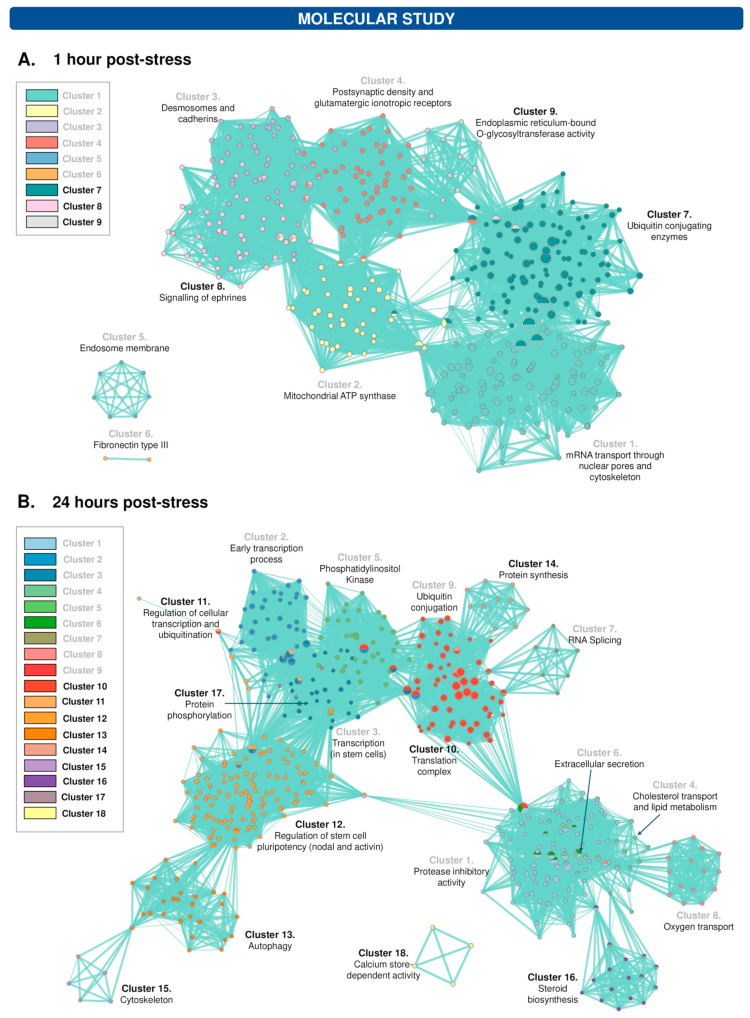
Acute stress induced an impairment of the hippocampal protein profile. Functional network in the context of 1 h (**A**) and 24 h (**B**) after the application of the acute stressor. The nodes (circles) indicate cellular functions or components in which the altered proteins are involved. Thus, each node has an identity in one or several clusters, represented by one or several colours. Clusters with grey font indicate underexpressed functions, and black font indicates overexpressed functions.

The hippocampal massive protein study revealed that stress caused changes in the protein profile 1 and 24 h after application, modulating the abundance of 28 proteins and 40 proteins, respectively. After selecting the networks formed by the set of underexpressed and overexpressed proteins (200 interactors) (Supplementary Information), the DAVID server was used to perform the functional enrichment study for each cluster of the networks (Table 4 and Table 5). One hour after the application of the acute stressor, functions associated with cell division; the cytoskeleton, mainly in relation to desmosomes and cadherins; and glutamatergic synapses were underexpressed. In addition, the processes of ubiquitination, cell differentiation, neurogenesis, and cell migration and O-glucosyl transferase activity were overexpressed. On the other hand, 24 h after the application of the stressor, transcriptional regulation, lipid metabolism and transport, and phosphatidylinositol metabolism were underexpressed, and protein synthesis, regulation of cellular transcription and ubiquitination, and autophagy were overexpressed. Figure 4A,B shows the functional networks for the molecular contexts at 1 and 24 h after the application of the acute stressor, respectively. This functional study was validated by Western blotting (Figure 3B) for both under- (1 h: GLUR7/GRIK3; 24 h: Pi4k2a; Figure 3C,D) and overexpressed (1 h: UBE2H; 24 h: Smad3; Figure 3E,F) proteins. The results of Western blotting (obtained by t-student) are shown in Figure 3G.

### 3.9. The Microglia of the Dentate Gyrus Responded with Morphological Changes after Exposure to WIRS

**Figure 5:**
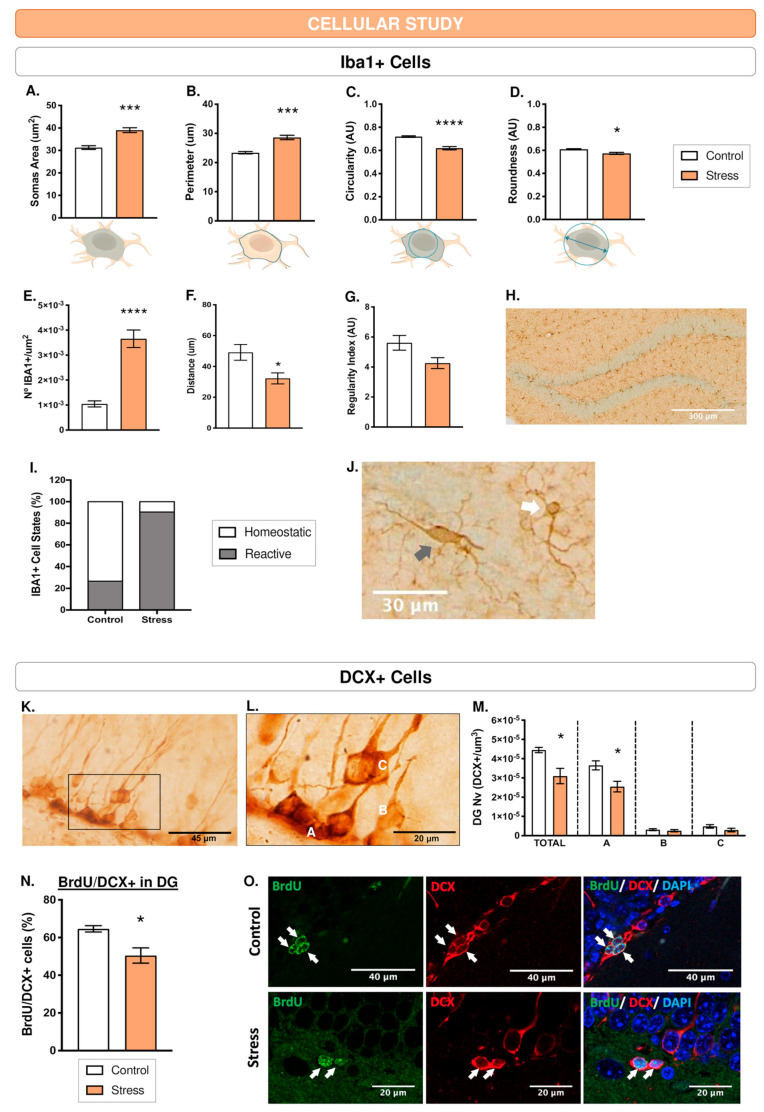
Acute stress induced cellular changes in the DG. (**A**–**J**) Study of microglia (Iba1+ cells) in terms of soma morphology parameters (**A**–**D**) and distribution parameters (**E**–**G**). (**H**) Panoramic micrograph of the hippocampal DG using anti-Iba1. (**I**) Morphological activation phenotype clustering: homeostatic and reactive. (**J**) Representative morphologies for clusters indicated in (**I**): homeostatic state (white arrow) and reactive to acute WIRS-type stress (dark arrow). (**K**) Labelling of DCX+ cells in the DG. (**L**) Detail in (**K**) showing classification of DCX+ cells following the maturity grading criteria described in the text (A, B and C, in white font). (**M**) DCX+ cell density in the DG. (**N**) Representation of the percentages of BrdU/DCX+ cells in the SGZ/CL of the DG. (**O**) Confocal microscopy images showing the presence of BrdU/DCX+ cells in the SGZ (subgranular zone) of the control and stressed groups. * *p* ≤ 0.05; *** *p* ≤ 0.005; **** *p* ≤ 0.0005 Control vs. Stress using Student’s *t* tests.

WIRS induced an increase in the area (t(8) = 1.19, *p* < 0.005; Figure 5A) and perimeter (t(8) = 1.16, *p* < 0.005; Figure 5B) and a decrease in the circularity (t(8) = −0.89, *p* < 0.0005; Figure 5C) and roundness (t(8) = −0.52, *p* < 0.05; Figure 5D) of the microglial soma. Furthermore, the Iba1+ cell density increased significantly in response to the stressor (t(8) = 0.18, *p* < 0.0005; Figure 5E) and the distance between the closest Iba1+ cells decreased (t(8) = −0.84, *p* < 0.05; Figure 5F), but the regularity index (RI) did not change (t(8) = 0.64, *p* > 0.05; Figure 5G).

In addition, hierarchical clustering studies revealed that microglia were grouped into two categories: Pattern 0 (smaller area, higher circularity, lower density and greater distance), which has usually been associated with a state of lower microglial stimulation or homeostatic conditions, and Pattern 1 (larger area, lower circularity, higher density and shorter distance), which is associated with stress-responsive microglia [38,39,42]. In response to WIRS, the results revealed that 90.48% of microglia corresponded to Pattern 1 (stress-responsive group), compared to 9.52% with Pattern 0 (homeostatic group) (Figure 5I,J).

### 3.10. WIRS Decreased the Number of DCX+ Cells

Acute stress decreased the total density of DCX+ cells in the DG (t(7) = 2.75, *p* < 0.05), particularly type A cells that present a lower degree of maturity (t(7) = 3.06, *p* < 0.05). The analyses showed no effect of acute stressors on type B (t(7) = 0.27, *p* > 0.05) and C cells (t(7) = 1.05, *p* > 0.05) (Figure 5L,M).

Similar to other studies [38,40,41,42,43], we found that most of the new cells belonged to the neuronal lineage; however, this percentage was lower in the stress group (Control: 60.64%; Stress: 50.49%), showing a decrease in the number of neurons after acute stress compared to the control group (t(4) = 3.59, *p* < 0.05) (Figure 5N,O).

The summary of all cell study results (analysed by *t*-student) are shown in Table 6.

## 4. Discussion

Witnessing an event that is perceived as life-threatening is often accompanied by intense fear, horror, and helplessness, which can lead to the development of post-traumatic stress disorder (PTSD). PTSD is a heterogeneous disorder in which numerous subtypes have been described depending, among other factors, on the type of stressor that provokes the disorder particularly human-made trauma and natural disasters [5]. This is a serious health concern that is associated with comorbidity, functional impairment, and increased mortality with suicidal ideations and attempts [44]. It is currently a common disorder, possibly due to social and climatic changes. However, the core neurobiological mechanisms of this disorder remain elusive, which could explain why the vast majority of PTSD patients displayed pharmacological unresponsiveness [45]. A more precise and neurobiologically based understanding will allow for increasingly effective, efficient, and personalized treatments. In this sense, PTSD requires a multidimensional approach that examines multiple biological levels (behavioral, molecular, cellular, etc.).

The aim of this work was to understand how exposure to acute and intense stress leads to a complex interplay of proteomic changes in the hippocampus, which plays a key role in the pathophysiology of PTSD [46]. In this sense, several high-profile traumatic events may led to development of PTSD, a heterogeneous disorder in which numerous subtypes have been described depending, among other factors, on the type of stressor that provokes the disorder particularly human-made trauma and natural disasters [5].

For this purpose, we have characterized the neurobiological profile of the animals subjected to acute stress protocol, WIRS, which mimics brief, intense, threatening experiences reminiscent of a natural catastrophe such as a flood with lasting affective consequences [19,47]. Therefore, we first studied the behavioral changes, then we studied the response of the HHA axis to acute and intense stress. We subsequently tried to understand how exposure to acute and intense stress leads to a complex interplay of proteomic changes in the hippocampus, which plays a key role in the pathophysiology of PTSD [46]. Finally, we performed a validation of the results obtained by massive protein analysis by Western blot, determination of hippocampal cytokines and cellular studies.

The results revealed that the application of WIRS results in behavioural and hormonal responses reminiscent of some changes observed in humans with PTSD. PTSD onset is associated with a clear triggering event, particularly a traumatic experience. In our study, animals subjected to this intense stressful experience showed anhedonic behaviour, which was observed the day after the application of the stressor and maintained even 6 days later. Moreover, an aversive state and negative emotional behaviours have been observed in stressed animals. In addition, in stressed animals, an alteration of the HHA axis reminiscent of that observed in people with PTSD was observed. the study of early molecular changes initiated following the stressor revealed increased inflammation and decreased neuroplasticity in the hippocampus that was corroborated by cellular studies.

From a behavioural point of view, anhedonia, which models clinical reports of emotional numbing in patients with PTSD [48], is a prevalent and consequential characteristic used for the diagnosis of PTSD [49]. Its occurrence may predict greater PTSD severity and long-term chronicity of symptoms [50] and may also be related to psychiatric comorbidity, illness trajectory and functional outcomes [51,52,53].

In animals subjected to WIRS, anhedonic response was associated with shorter time spent in each of the open arm entries in the EPM and with a higher frequency of grooming in OFT. These data together could be related to greater anxiety and discomfort experienced by stressed animals. In fact, increased anxiety-like behaviour in the EPM has been observed in animal models of PTSD [28,29,30], and patients with PTSD suffer from anxiety [52]. An increased frequency of grooming has been observed in animal models of PTSD [54] and rats administered an antagonist of the orexin A/B receptor, a peptide found to be reduced in people with PTSD [55]. These behaviours have been related to aversive state and negative emotional behaviours [56], showing that stressed animals are less resilient to developing PTSD-associated symptoms, as revealed by PCA.

Additionally, stressed animals exhibited a decrease in CORT levels (28 h post-stress) after an initial high release (10–30 min after stress application). PTSD patients present abnormalities in HPA axis regulation [57]. In this regard, in humans, a massive surge of cortisol may be the result of exposure to a traumatic event that is hypothesized to trigger the disorder [9] and, among other symptoms, has been associated with the development of anhedonia [58]. However, following this initial hypercortisolaemia, numerous studies have reported a hypoactive HPA axis in patients with PTSD [9] that may contribute to the maintenance of symptoms [59]. In this respect, dysregulation of this hormone can have adverse effects, and counterintuitively, the administration of cortisol after victims have suffered trauma reduces the eventual development of core PTSD symptoms [60,61]. Our findings of lower corticosterone levels 28 h after WIRS may support the construct validity of our model.

Identification of the molecular basis of stress-induced pathology is critical for devising means of mitigating the severity of traumatic disorders. Studying the immediate and early responses to trauma may provide clues to the way PTSD develops and may improve the understanding of the neurobiological mechanisms involved in the onset of PTSD, opening the opportunity for possible preventive treatments. Proteomics is a promising strategy for characterizing the neurobiological mechanisms underlying the development of PTSD [14]. The data revealed that stress induced an extensive change in the hippocampal proteome, modifying the abundance of 28 and 40 proteins after 1 h and 24 h of stress application, respectively. Protein expression assayed by Western blotting showed a comparative direction of expression with the protein determination performed by Orbitrap. Among other modified proteins, data, confirmed via Western blotting, revealed a reduction in the levels of phosphatidylinositol 4-kinase (PI4K) and an increase in the levels of Smad3 24 h after stress. Both proteins have been linked to the development of inflammation. PI4K is a lipid kinase that catalyses the phosphorylation of PtdIns to produce PI4P, which has an important influence on the progression of many diseases and biological functions, revealing that it is closely related to the occurrence and development of inflammation [62]. Moreover, impairment of Smad3, specifically TGFβ-Smad3 signalling, could reduce the capability of microglia to deal with injury, inducing progression towards a more inflammatory state [63]. Furthermore, 1 and 24 h after acute stress, GluR7 levels were downregulated and upregulated, respectively. This receptor has been implicated in neuroplasticity and in several neuropsychiatric conditions, such as PTSD [64,65,66], and glutamatergic systems are involved in the reinforcement system [67], which could be related to the anhedonia observed in stressed animals.

Nevertheless, understanding the protein–protein interaction complexes and information-processing capabilities of signal transduction networks and how those networks are disrupted in disease may illuminate the amazing breadth and complexity of the signal transduction pathways that cells employ to respond to stress. Functional enrichment data using DAVID and subsequent functional clustering revealed that the affected stress-associated pathways included metabolic, mitochondrial function, gene expression, signalling and synaptic communication pathways.

Although the overall interpretation of the observed changes in the protein profile is complex, taken together, the data indicate that an acute stressor leads to an imbalance in the cellular transcription and translation machinery. In addition, many functional pathways involved in neurogenesis and inflammation are affected. Thus, 1 h after stress, a reduction in signalling pathways involved in cytoskeleton formation, cell division (Clusters 1 and 3), ATP-mediated cell division (Cluster 2), and the glutamatergic system (Cluster 4), and the upregulation of those involved in angiogenesis (Cluster 8), was observed. All these pathways have been implicated in hippocampal neurogenesis [68,69,70,71,72]. On the other hand, the reduction in proteins involved in ATP regulation and ATP synthase may be linked to oxidative stress and may be implicated in the occurrence of inflammation [73]. Moreover, ATP is the primary chemoattractant released from the damaged tissue, which regulates microglial branch dynamics and mediates a rapid microglial response to injury [74].

Within 24 h of stress, an imbalance was observed in pathways involved in transcription (Clusters 2, 3, and 11), translation (Clusters 7, 10 and 14), and posttranslational changes (Clusters 5, 9, and 17), as well as an increase in pathways involved in lipid metabolism (Cluster 4) and autophagy (Cluster 13). Autophagy has emerged as a regulator of microglial functions and is related to the regulation of metabolic status and inflammation [75]. In addition to these alterations, 2 h of acute stress application also affected signalling pathways that are dysregulated in neurodegenerative diseases. In summary, the data obtained at the molecular level may explain some of the behavioural effects induced by stress. However, further studies are needed to link the behavioural and hormonal changes and the findings more precisely with proteomics techniques. To the best of our knowledge, this is the first study to perform a massive analysis of proteins in the hippocampus following acute stress (WIRS).

With the aim of confirming the effect of acute WIRS on the hippocampal proteome, we studied the cellular changes induced by WIRS. Our data revealed that acute stress induced an increase in microglial number accompanied by a reduction in cell spacing and morphological changes in the DG. These morphological changes in the cellular soma, induced by acute stress, may indicate the presence of hypertrophic microglia [76]. Enlarged microglial somas are typically described as the first stage of microglial response to pathological situations and have been observed after several types of brain damage [77,78,79]. Hierarchical clustering analysis supports these data, with a high proportion of microglia classified as responding to acute stress compared to the homeostatic microglia observed preferentially in control animals. These data reveal that hippocampal microglia respond to WIRS. These results are in line with most studies that have demonstrated that stress promotes significant structural remodelling of microglia and may enhance the release of proinflammatory cytokines from microglia [80]. In fact, WIRS induced a nonsignificant increase in hippocampal IL-6, TNF-α, and IFN-γ levels 1 h after stressor application. At the 24 h assessment, IL-6 levels remained elevated, but TNF-α and IFN-γ levels did not. Although the details are not known, the response of microglia to acute stress and the increase in inflammatory cytokines could be mediated, among other mechanisms, by an increase in CORT levels. Several studies have provided a strong connection between an increase in CORT levels and an increase in microglia response to stress, with both in vivo and in vitro studies revealing that an excess of CORT increases neuroinflammation [81] and changes microglial function [82]. As mentioned above, WIRS can be considered an intense stress that dysregulates the hypothalamic–pituitary–adrenal axis, which could explain the results. The increased inflammation is in line with other studies in both animal models and humans in which it has been suggested that inflammation is not only associated with PTSD but also can play an important role in its pathogenesis and pathophysiology [83].

Adult hippocampal neurogenesis, which in recent years has been increasingly linked to the stress response, represents a remarkable form of neuroplasticity [84]. Acute and intense stress is also known to reduce the level of hippocampal neurogenesis [85]. Similarly, fewer DCX+ immature cells in the infrapyramidal subdivision of the dentate gyrus have also been observed after a chronic stress procedure [86]. From a clinical perspective, in individuals subjected to chronic or traumatic stress, adult hippocampal neurogenesis disruption may be involved in inappropriate evaluation and regulation of emotions, potentially resulting in affective neuropathologies. Moreover, a decrease in adult hippocampal neurogenesis may be a risk factor for the development of PTSD-related symptoms, since the ablation of neurogenesis affects stress susceptibility [87]. In contrast, in animal models, neurogenesis enhancers improve PTSD-like behaviour [88]. A reduction in the production of new neurons and impairment in the differentiation of new cells into neurons have been observed after WIRS. Thus, stress reduced both BrdU/DCX double-labelled cells and the density of DCX+ cells in the DG, particularly DCX+ cells of more immature morphology (type A) and intermediate morphology (type B). Acute stress had a greater impact on the more undifferentiated cells in the DG.

PTSD requires a multidimensional approach that examines different causes and consequences at different biological levels (behavioural, molecular, biochemical and cellular). Overall, our data revealed, for the first time, that WIRS exposure induces long-term sustained anhedonia, discomfort symptoms, and alterations in the hippocampal proteomic profile with an imbalance in favour of increased inflammation and decreased plasticity, as confirmed by cytokine and immunocytochemical determination. Future studies are needed to determine whether there are molecular, histological and behavioural differences in models that mimic some of the characteristics of natural disasters or human-made traumas to understand neurobiological differences. In addition, further studies are needed to understand sex differences in the neurobiological response to acute and intense stress. However, despite the limitations, this study has allowed us to further characterise the neurobiological alterations induced by WIRS. Understanding early changes that could be responsible for neurobiological changes after exposure to traumatic events may be the starting point that allows increasingly efficient, impactful, and individually tailored treatments.

## 5. Conclusions

In conclusion, our data revealed that following an acute stressor, particularly an intense stressor, many changes are initiated that provide valuable insights into the mechanisms involved in the stress response. Thus, the results show that stress exposure restricted to 2 h may induce anhedonia, a symptom associated with increased severity and chronicity of the disease, higher anxiety, and lower CORT levels after an initial increase due to stress, indicating that it may be a good model for some of the symptoms associated with PTSD. Moreover, the expression profile of hippocampal proteins was drastically altered after acute stress. Proteins that represent intermediate phenotypes for the disease provide insight into how stress impacts at the cellular level by inducing changes. The protein profile was deregulated in favour of increased inflammation and reduced neuroplasticity, which was validated by histological studies and cytokine determination. These sustained stress-related changes suggest a mechanism whereby acute stress affects the extended stress response and may contribute to explaining how limited exposure to acute stress results in the pathogenesis of stress-related disorders, particularly PTSD, and could be useful as a possible model of natural disaster-induced PTSD.

## Figures and Tables

**Table 1:** Summary of the experimental blocks used.

Block	Stress (WIRS)	CORT (ELISA)	Behavioural Test (OFT, SPT, EPM, TST)	Molecular (Cytokines and Proteomic Profiles)	Cellular (Iba1+, BrdU+, DCX+)
I	+	+	+	−	−
II	+	+	−	+	-
III	+	+	−	−	+

Note: The + sign indicates that the test was performed and the − sign indicates that this procedure was not carried out for the development of the experiment.

**Table 2:** Results of corticosterone determinations.

Study	Context	Statistic	*p*-Value
Behavioural	90 min after finishing behavioural procedures in both groups	Repeated-measures ANOVA: F(2, 26) = 14.30	*p* < 0.0005 LSD: *p* < 0.005
Molecular	10 min post-stress 30 min post-stress 1 h post-stress 24 h post-stress	t(33) = −6.51 t(32) = −3.87 t(13) = −1.05 t(19) = −0.003	*p* < 0.0005 *p* < 0.005 *p* > 0.05 *p* > 0.05
Histological	24 and 28 h post-stress	Repeated-measures ANOVA (for the environmental treatment factor): F(1, 4) = 11.83	*p* < 0.05 LSD: *p* < 0.05

**Table 3:** Results of behavioral tests.

Behavioural Test	Variable	Statistic	*p*-Value
OFT	**Distance**: periphery centre total **Time**: periphery centre **Velocity**	t(26) = −0.27 t(26) = −0.09 t(26) = −0.27 t(26) = −0.56 t(26) = −0.40 t(26) = −0.15	*p* > 0.05 *p* > 0.05 *p* > 0.05 *p* > 0.05 *p* > 0.05 *p* > 0.05
SPT	24 h post-stress	Repeated-measures ANOVA: F(1, 26) = 14.54	*p* < 0.005 LSD: *p* < 0.0005
	6 days after the end of the stressor	Repeated-measures ANOVA performed on the additional F(2, 22) = 5.94	*p* < 0.01 LSD: *p* < 0.005
EPM	**Anxiety index** **Time**: centre open arms closed arms **Frequency entry:** centre open arms closed arms **Time/frequency (open arms**)	t(26) = 0.88 t(26) = 0.91 t(26) = −0.42 t(26) = 0.91 t(26) = −0.52 t(26) = −0.96 t(26) = −0.18 t(26) = 0.06	*p* > 0.05 *p* > 0.05 *p* > 0.05 *p* > 0.05 *p* > 0.05 *p* > 0.05 *p* > 0.05 *p* > 0.05
TST	**Immobility** **Energy** **PM**	t(26) = 1.58 t(26) = −0.28 t(26) = −0.17	*p* > 0.05 *p* > 0.05 *p* > 0.05

**Table 4:** Functional network clusters in the context of 1 h after the application of the acute stressor.

Type of Expression	Cluster	Information *
Underexpression	Cluster 1	Transport of messenger RNA (mRNA) through nuclear pores (40.28) Kinetochore, chromosome segregation and centromere (33.29) Nuclear membrane (9.23)
	Cluster 2	Mitochondrial ATP synthase complex (22.53) Ion transport, Huntington’s disease, Alzheimer’s and Parkinson’s (19.03) ATP synthesis (14.29) Myelin sheath (2.64)
	Cluster 3	Desmosomes (10.7 y 6.13) Cadherins and calcium binding (7.71) Regulation of cell adhesion involved in cardiac muscle contraction (6.38)
	Cluster 4	Glutamatergic ionotropic receptor and postsynaptic density (7.52) Glutamatergic synapses (5.98) Membrane-associated guanylate kinase (4.01)
	Cluster 5	Endosome membrane (2.09)
	Cluster 6	Fibronectin type III (1.86)
Overexpression	Cluster 7	Ubiquitin-conjugating enzymes (30.4) HECT domain, ubiquitin-binding enzyme-related domain (9.79)
	Cluster 8	Ephrines signalling (12.08) Ras and PI3K/Akt signalling (6.15) Cell differentiation and neurogenesis (3.27) Cell migration and angiogenesis (3.13) Development of dendritic spines, VEGFR and semaphorins (2.53)
	Cluster 9	Endoplasmic reticulum-bound O-glycosyltransferase activity (3.38)

* Note: the enrichment score assigned by the DAVID server to the biological function prediction is shown in parenthesis. This score indicates the importance of a set of genes, with respect to the total list of genes for that cluster.

**Table 5:** Functional network clusters in the context of 24 h after acute stressor application.

Type of Expression	Cluster	Information *
Underexpression	Cluster 1	Protease inhibitor (7.73) Blood coagulation and haemostasis (6.23) Golgi Complex and ER (4.87) Cholesterol metabolism (4.64)
	Cluster 2	Early transcription process (35.19 and 10.6)
	Cluster 3	Gene transcription (13.4) Gene transcription related to stem cell population maintenance (4.2) Exonuclease activity (3.97) Alternative splicing (3.87)
	Cluster 4	Cholesterol transport (6.83) Lipidic metabolism (4.22) Lipoprotein metabolism (3.9) Lipid transport (3.81) Disulfide bonding and glycosylation (3.54) Extracellular región (3.42) Lipid homeostasis (2.58)
	Cluster 5	Phosphorylation (7.56) Phosphatidylinositol Kinase (PIPK) (5.47) PI3K activity (4.76) Synthesis of phosphatidylinositols in the Golgi membrane (4.08)
	Cluster 6	Extracellular secretion (2.91)
	Cluster 7	RNA splicing (2.52)
	Cluster 8	Oxygen transport (4.98)
	Cluster 9	Ubiquitin conjugation (1.8)
Overexpression	Cluster 10	Translation initiation complex (70.1) Initiation of protein synthesis (27.85) Ribosomal proteins (27.51) Proteasome initiation complex (4.77) RNA binding to proteins (0.99)
	Cluster 11	Regulation of cellular transcription and ubiquitination (8.99) Endosomal transport (2.12) Cytoplasmic vesicle (1.91)
	Cluster 12	Signalling pathway regulating stem cell pluripotency (nodal and activin) (13.92) TGF-β signalling (9.61 and 4.53)
	Cluster 13	Autophagy (5.13) Autophagy regulation (4.81)
	Cluster 14	Protein synthesis (2.97)
	Cluster 15	Protein binding and cytoskeleton, in general (3.27)
	Cluster 16	Steroid biosynthesis (3.91)
	Cluster 17	Protein phosphorylation (1.62)
	Cluster 18	Calcium store-dependent activity (2.47)

* Note: the enrichment score assigned by the DAVID server to the biological function prediction is shown in parenthesis. This score indicates the importance of a set of genes, with respect to the total list of genes for that cluster.

**Table 6:** Results of the cellular studies.

Study	Variable	Statistic	*p*-Value
Iba1+ Cells	Area	t(8) = 1.19	*p* < 0.005
	Perimeter	t(8) = 1.16	*p* < 0.005
	Circularity	t(8) = −0.89	*p* < 0.0005
	Roundness	t(8) = −0.52	*p* < 0.05
	N° Iba1+/um^2^	t(8) = 0.18	*p* < 0.0005
	Distance	t(8) = −0.84	*p* < 0.05
	RI	t(8) = 0.64	*p* > 0.05
DCX+ Cells	Total DG	t(7) = 2.75	*p* < 0.05
	Type A	t(7) = 3.06	*p* < 0.05
	Type B	t(7) = 0.27	*p* > 0.05
	Type C	t(7) = 1.05	*p* > 0.05
	BrdU/DCX+ Cells	t(4) = 3.59	*p* < 0.05

## Data Availability

The data presented in this study are deposited and made publicly available in: Dataset Orbitrap Raw-Data Stress Hippocampus(1 h–24 h): https://doi.org/10.24310/riuma.26238.

## Supplementary Materials

The following supporting information can be downloaded at: https://www.mdpi.com/article/10.3390/cells12182290/s1.
