# Supplementary material for: Unveiling the Secrets of the Stressed Hippocampus: Exploring Proteomic Changes and Neurobiology of Posttraumatic Stress Disorder
Source: Cells. 2023 Sep 15;12(18):2290. doi: 10.3390/cells12182290 (PMC10527244; doi:10.3390/cells12182290)
Supplement: Supplementary file 1 [file cells-12-02290-s001.zip › cells-2501146-supplementary.pdf]

SUPPLEMENTARY INFORMATION

Unveiling the Secrets of the Stressed Hippocampus: Exploring Proteomic Changes and Neurobiology of Posttraumatic Stress Disorder

Andrea Nieto-Quero, María Inmaculada Infantes-López, Emma Zambrana-Infantes, Patricia Chaves-Peña, Ana L Gavito, Jose Munoz-Martin, Sara Tabbai, Javier Márquez, Fernando Rodríguez de Fonseca, María Inmaculada García-Fernández, Luis J Santín, Carmen Pedraza*, Margarita Pérez-Martín*

*Corresponding author

Supplementary Information includes:

- Corticosterone measurement
- Information extracted from MCODE (1 hour post-stress)
- Information extracted from MCODE (24 hours post-stress)
- Western Blot
- Immunolabeling assays
- Quantification and morphological analysis for Iba1+, DCX+ and BrdU/DCX+
- Basal locomotor and exploratory activity measured by OFT
- WIRS did not affect the parameters assessed by the EPM
- Figure S1: Complementary behavioural results for the OFT and EPM tests
- Figure S2: Western blot membranes

Corticosterone measurement

Basal and post-stress blood samples, from the lateral tail vein, were collected in EDTA tubes at 9:00 am and 3:00 pm. The final samples were collected from the right atrium of each animal at the time of sacrifice. The blood sample tubes were centrifuged (10 min, 6000 rpm, 4°C), and the supernatant (plasma for basal and post-stress samples and serum for final samples) was stored at -80 °C. Plasma and serum corticosterone levels were determined using a commercially available Enzyme Immunoassay Kit, sensitivity ca. 27.0 pg/ml, following the manufacturer's instructions (Assay Designs/Stressgen, Ann Arbor, Michigan, USA).

Information extracted from MCODE (1 hour post-stress)

In the network for the set of underexpressed and overexpressed proteins, after 1 h of the stressor exposure, MCODE identifies a total of 9 clusters, among them 6 are related to underexpression (Table S1), and 3 to overexpression (Table S2). For each cluster we have information on the number of nodes, the number of interactions, and the cluster score (a higher score indicates stronger connections between nodes). This information can be consulted in the following.

Table S1. Information on the clusters that form the network for the 1-hour context after exposure to the stressor, and that are associated with significantly underexpressed proteins.

**No. Cluster**	**1**	**2**	**3**	**4**	**5**	**6**
**No. of nodes in the cluster**	58	21	10	7	4	3
**No. of interactions**	1159	206	45	21	6	3
**Cluster score**	40.7	20.6	10.0	7.0	4.0	3.0

Table S2. Information on the clusters that form the network for the context of 1 hour after stressor exposure, and which are associated with significantly overexpressed proteins.

**No. Cluster**	**7**	**8**	**9**
**No. of nodes in the cluster**	79	15	6
**No. of interactions**	3081	80	15
**Cluster score**	79.0	11.4	6.0

These 9 clusters are formed, specifically, by the proteins encoded by the following genes:

<u>Cluster 1</u>

Cenpe
Ipo8
Nup153
Nup50
Nde1
Ccnb1
Nup93
Pom121
Ndc1
Tpr
Xpo1
Nxf7
Nup107
Gle1
Aaas
Seh1l
Ahctf1
Nup62
Ndel1
Ndc80
Cenpl
Bub1
Pafah1b1
Nup155
Dsn1
Cenpf
Nup43
Nup133
Sec13
mCG_49183
Nxf2
Nxf1
Ube2i
Incenp
Nup188
Nup98
Rae1
Bub1b
Ranbp2
Smc3
Nup210
Nup37
Nupl1
Nup54
Sgol2
Nup205
Nup88
Nup160
Mad2l1
Bub3
Rangap1
Cenpc1
Sgol1
Nup85
Zwilch
Nuf2
Ska2
Nup214

<u>Cluster 2</u>

Atp5g1
Atp5f1
Atp5o
Ndufs7
Atp5a1
Atp5g3
Gm10221
Atp5h
mt-Atp6
Atp5s
Atp5g2

Atp5c1
Atp5k
Uqcr11
Atp5e
Atp5d
Atp5j2
Atp5j
Gm10175
Gm5426
Atp5b

Cluster 3

Pkp4
Dsp
Dsg2
Dsc3
Dsg4
Dsg1a
Jup
Dsc1
Dsg3
Dsc2

Cluster 4

Grik3
Dlg1
Dlg4
Grik2
Dlg3
Grik5
Grik4

Cluster 5

Pikfyve
Fig4
Mcoln1
Vac14

Cluster 6

Cntn6
Ptprg
Chl1

Cluster 7

Uba5
Hectd2
Fbxl3
Ube2g1
Cdc34-ps
Cdc34
Nedd4l
Arel1
Smurf2
Ube2e1
Pja1
Fbxl15
Ube2z
Ube2s
Wwp1
Cul7
Ube2r2
Herc6
Trim21
Atg7
Fbxo40
Trim71
Ubr2
Ubr1
Ube2o
Hace1
Ube3a
Rlim
Uba6
Cul1
Lnx1
Ube2g2
Rbck1
Stub1
Trim69
Traf7
Trip12
Ube2b
Ube2j1
Trim63
Mgrn1
Ube2d3
Skp1a
Ube2a
Itch
Ubox5
Ube2d2a
Fbxl19
Nedd4
Uba7
Smurf1
Ube2j2
Ube2c
Ube2d1
Ube4a
Rbx1
Ube2k
Uba1
Ube2u
Ube2v2
Uba3
Pja2
Ube2h
Rnf25
Ube2e3
Ube2q1
Park2
Rnf41
Fbxo32
Trim41
Hecw2
Ubc
Ube2e2
Ube2m
Ube2q2
Ube2l6
Cul3
Huwe1

Cluster 8

Efna4
Epha10
Efnb3
Efnb1
Efna2
Efna3
Fyn
Pak2
Efna5
Efnb2
Pak3
Nck2
Efna1
Ephb3
Rac1

Cluster 9

Dag1
Pomk
Pomgnt2
Pomt1
Pomt2
B3galnt2

Information extracted from MCODE (24 hours post-stress)

In the network for the set of underexpressed and overexpressed proteins, after 24 hours of stressor exposure, MCODE identifies a total of 18 clusters, of which 9 are related to underexpression (Table S3), and 9 to overexpression (Table S4). For each cluster we have information on the number of nodes, the number of interactions, and the cluster score. This information can be found in the following.

Table S3. Information on the clusters forming the network for the 24-hour context after stressor exposure, and which are associated with the significantly underexpressed proteins.

No. Cluster	1	2	3	4	5	6	7	8	9
No. of nodes in the cluster	31	20	11	9	9	5	3	3	3
No. of interactions	465	189	55	35	33	7	3	3	3
Cluster score	31.0	19.9	11.0	8.8	8.3	3.5	3.0	3.0	3.0

Table S4. Information on the clusters that form the network for the 24-hour context after stressor exposure, and which are associated with significantly overexpressed proteins.

No. Cluster	10	11	12	13	14	15	16	17	18
No. of nodes in the cluster	43	11	14	9	4	4	4	3	3
No. of interactions	896	55	69	36	6	6	6	3	3
Cluster score	42.7	11.0	10.6	9.0	4.0	4.0	4.0	3.0	3.0

These 18 clusters are made up of proteins encoded by the following genes:

Cluster 1

Kng1
Il6
Cst3
Ahsg
Fn1
C3
Kng2
Fgg
Fga
Serpina1a
Apob
Igfbp1
Apoe
Serpind1
Serpina1e
Pcsk9
Itih2
C4b
Serpina1d
Apoa2
Trf
Apoa5
Cp
Serpinc1
Apoa1
Afp
App
Proc
Spp2
Alb
Serpina1b

Cluster 2

Ccnk
Supt6
Polr2f
Ctdp1
Supt4a
Tcea1
Gtf2f2
Polr2d
Ccnt1
Polr2b
Gtf2f1
Cdk12
Ctr9
Polr2k
Iws1
Cdk9
Polr2c
Polr2g
Cdk7
Supt5

Cluster 3

Cnot7
Cnot6l
Rqcd1
Cnot2
Cnot8
Cnot10
Cnot1
Cnot4
Cnot11
Cnot3
Cnot6

Cluster 4

Pltp
Pon1
Scarb1
Lpl
Lcat
Mttp

Apoc2
Ldlr
Abca1

Cluster 5

Pi4ka
Sacm1l
Pi4kb
Pip5k1b
Pik3c3
Pip5k1c
Pikfyve
Pip5k1a
Pi4k2a

Cluster 6

Apoc3
A2m
Apom
Hp
Apoh

Cluster 7

Hnrnpu
Snrpb
Ybx1

Cluster 8

Hbb-bh1
Hba-a2
Hbb-y

Cluster 9

Skp2
Ubac1
Rnf123

Cluster 10

Rps18
Rps5
Rps11
Eif2s3y
Rps27l
Eif3d
Rps23
Rps8
Rps28
Eif3a
Rps7
Rps3
Rps15a
Eif3h
Rps3a1
Eif3m
Rps16
Rps21
Eif3c
Eif2s3x
Eif3f
Eif3i
Eif3g
Rps9
Gnb2l1
Rps17
Eif3k
Eif3l
Fau
Eif2s1
Rps25
Eif3j1
Eif3b
Eif3e
Rps6
Rps13
Eif4a1
Rps27a
Eif3j2
Rps27
Rps19
Eif4b
Rps14

Cluster 11

Ccdc22
Commd9
Commd8
Commd6
Commd3
Commd7
Commd4
Commd1
Ccdc93
Commd10
Commd2

Cluster 12

Smad2
Foxh1
Smad4
Acvr1c
Acvr1b
Zfyve9
Tgfb1
Ski
Acvr2a
Smurf2
Smad3
Tgfbr1
Acvr2b
Smad7

Cluster 13

Map1lc3b
Gabarap
Becn1
Uvrag
Pik3c3
Rubcn
Pik3r4
Gabarapl1
Map1lc3a

Cluster 14

Pet112
Nars
Qrsl1
Nars2

Cluster 15

Ppp4c
Smek2
Smek1
Ppp4r2

Cluster 16

Dhcr24
Lss
Sc5d
Dhcr7

Cluster 17

Cdk2
Ccnt1
Cdk9

Cluster 18

Orai2
Stim1
Stim2

Western Blot

The hippocampi were homogenised in RIPA lysis buffer with protease and phosphatase inhibitors cocktail. The protein concentration of each sample was determined by the Bradford method (Bio-Rad protein assay, Bio-Rad, California, United States). Protein extracts ($2.3x10^3$µg/ml) were separated by 4-12% Criterion™ gel for protein electrophoresis (Bio-Rad, Madrid, Spain), and electrically transferred to nitrocellulose membranes (Bio-Rad, Madrid, Spain) using the Trans-Blot® Turbo™ transfer system (Bio-Rad, California, USA). The membranes were blocked with 2% BSA for 1 hour. Subsequently, the membranes were incubated overnight with the primary antibodies: mouse anti-β-actin (1:1000; Thermo Scientific, ref: MA1-140); mouse anti-Smad3 (1:200; Santa Cruz, ref: sc-101154); rabbit anti-GLUR7 (1:200; Thermo Scientific, ref: PA5-77435); rabbit anti-Pi4k2a (1:1000; Thermo Scientific, ref: PA5-15275); rabbit anti-UBE2H (1:500; Thermo Scientific, ref: PA5-96648). After washing with TBS-T, they were incubated for 1 hour with the secondary antibodies: anti-mouse-HRP conjugated (1:10000; Promega, ref: W4021); anti-rabbit-HRP conjugated (1:10000; Promega, ref: W4011). Immunocomplexes were visualised using a peroxidase chemiluminescence substrate (Clarity Max™ Western ECL Substrate, Bio-Rad Laboratories, Cressier, Switzerland) and immunoreactivity was detected using the ChemiDoc XRS system (Bio-Rad Laboratories, Cressier, Switzerland). Finally, protein quantification was performed with ImageJ software (Rasband, W.S., ImageJ, National Institutes of Health, Bethesda, Maryland, USA). Each protein band was normalised to the β-actin levels of the same membrane.

Immunolabeling assays

Once the mice, of experimental block III, were anesthetized with sodium pentobarbital 200 mg/ml, the brains were collected after the sacrifice with PBS (pH = 7.4) and 4% paraformaldehyde. After 48 h of post-fixation in 4% paraformaldehyde, brains were stored at -15ºC in cryoprotectant solution. Brains were sectioned on 40 µm coronal sections using a vibratome. Thus, for the set of slices used, there is approximately 240 µm between the closest sections rostro-caudally. First, endogenous peroxidase was inactivated by 10% methanol and 10% H_2O_2 in PBS for 45 minutes in the dark. Incubation in saturating solution for 45 minutes. Next, primary antibodies: rabbit anti-Iba1 (1:500; Wako, ref: 019-19741) and goat anti-DCX (1:200; Santa Cruz Biotechnology, ref: sc-8066) were used for a overnight incubation at 4°C. Followed by the correspondent biotynilated secondary antibodys: anti-rabbit (1:1000; Dako, ref: E0432) and anti-goat (1:1000; Dako, ref: E0466) for 1 hour and 30 minutes. This was followed by incubation with ExtrAvidin peroxidase (1:1000; Sigma-Aldrich, ref: E2886) for 1 hour in the dark. And finally, a revealed with 0.05% diaminobenzidine (DAB) and 0.03% H_2O_2 for up to 5 minutes in the dark. After each reagent and antibody treatment, three 10-minute with shaking washes with PBS were performed.

Otherwise, for a concrete study of new neurons proliferation, double positive cells were labelled following an immunofluorescence protocol. The tissue was treated with 2 N HCl at 37°C for 15 minutes. Then a saturation phase for 45 minutes was performed. Then primary antibodies: rabbit anti-DCX (1:600; Abcam, ref: ab18723) and rat anti-BrdU (1:1000; Accurate Chemical, ref: OBT0030) were used for a overnight incubation at 4°C. Followed by the correspondent secondary antibodies: Alexa Fluor 568 anti-rabbit (1:1000; Invitrogen, ref: A10042) and Alexa Fluor 488 anti-rat (1:1000; Invitrogen, ref: A21208) for 1 hour and 30 minutes in the dark. And finally, a treatment with DAPI 1X was used as nuclear marker. After each reagent and antibody treatment, three 10-minute with shaking washes with PBS were performed.

Quantification and morphological analysis for Iba1+, DCX+ and BrdU/DCX+

For the analysis of the microglia, tissue sections immunostained with anti-Iba1 containing the dentate gyrus (DG; Fig. 5H) (a mean of 6 sections by each animal) were scanned with a 20x objective using VS-ASW software linked to an Olympus VS120 virtual microscope (Olympus, Tokyo, Japan). Through subsequent image analysis with the free software program Fiji (Rasband, W.S., ImageJ, National Institutes of Health, Bethesda, Maryland, United States), each microglia was quantified and analysed. Morphometric parameters of the cell soma (area, perimeter, circularity and roundness) and distribution (density, distance and regularity index) were considered (Davis et al., 2017). Where each parameter refers to the following:

- Area: surface occupied by the soma (μm^2).
- Perimeter: length of the soma contour (μm).
- Circularity: parameter derived from the area and perimeter. Its value lies in the interval [0, 1], with 0 corresponding to a completely elongated soma, and 1 corresponding to a completely circular soma.
- Roundness: parameter derived from the area and major length of the soma. Its value lies in the interval [0, 1], where 0 is indicative if it contains very angular regions and 1 if it contains completely rounded regions.
- Density: number of Iba1+ cells quantified in relation to the selected area of the DG.
- Distance: space between the microglia analysed with respect to the microglia closest to it. The centre of the soma of each microglia is taken as the points to plot this distance. (μm).
- Regularity Index (RI): provides information on the uniformity of the distribution of microglia. This parameter is calculated as follows: Distance/ Standard deviation of the distances.

For DCX+ cells of DG (Fig. 5K, L) were quantified using a 100x objective of the Olympus BX53 microscope (Olympus, Tokyo, Japan) and equipped with the newCAST™ computerized system with Autodisector™ (Visiopharm, Hoersholm, Denmark). A mean of 6 sections by animal were quantified. To determine the density of the different types of neurons according to their degree of maturity, the following classification was made based on morphology: type A, proliferative cells or more immature cells without prolongations; type B, immature neurons with one prolongation; and type C, neurons with at least one branch in some of their prolongations extend into

the molecular layer (Brown et al., 2003) (Fig. 5L). The results obtained by this method provided us with a density value for the following formula:

$$N_V = \frac{\sum(Q - d)}{\sum(h\ x\ a_{fra})} ,$$

where N_V refers to cell density, ΣQ−d is the total number of positive cells for a specific type of labelling, and Σ(h x a_{fra}) is the quantification volume, where h is the depth of the optical dissector and a_{fra} is the area of the optical dissector.

Furthermore, to determine the possible new neurons (BrdU/DCX +) in a group, approximately 110 BrdU+ cells were quantified by animal using a confocal microscope (Stellaris 8, Leica, Wetzlar, Germany).

Basal locomotor and exploratory activity measured by OFT

The analysis revealed no significant differences between groups in spatio-temporal parameters: distances travelled in periphery ($t(26)=-0.27$, $p>0.05$), centre ($t(26)=-0.09$, $p>0.05$) and total ($t(26)=-0.27$, $p>0.05$) of the arena (Fig. 2A); time in the periphery ($t(26)=-0.56$, $p>0.05$) and centre ($t(26)=-0.40$, $p>0.05$) (Fig. 2B); and velocity ($t(26)=-0.15$, Fig. 2C). No significant differences were observed either, respect to the control group, regarding frequency and time (Fig. S1A, B in this document of Supplementary Information) of the behaviours evaluated: wall rearing (frequency: $t(26)=1.82$, $p>0.05$; time: $t(26)=1.68$, $p>0.05$), rearing (frequency: $t(26)=-0.30$, $p>0.05$; time: $t(26)=-0.24$, $p>0.05$), and grooming (frequency: $t(26)=-0.66$, $p>0.05$; time: $t(26)=-0.84$, $p>0.05$).

WIRS did not affect the parameters assessed by the EPM

Regarding the spatiotemporal parameters, no significant differences between treatments were observed: anxiety index ($t(26)=0.88$, $p>0.05$; Fig. 2E), time spent (centre: $t(26)=0.91$, $p>0.05$; open arms: $t(26)=-0.42$, $p>0.05$; closed arms: $t(26)=0.91$, $p>0.05$; Fig. 2F), frequency of entry (centre: $t(26)=-0.52$, $p>0.05$; open arms: $t(26)=-0.96$, $p>0.05$; closed arms: $t(26)=-0.18$, $p>0.05$; Fig. 2G), and the time/frequency ratio on open arms ($t(26)=0.06$, $p>0.05$; Fig. 2H). WIRS also did not change the ethological parameters assessed: wall rearing (frequency: $t(26)=-0.53$, $p>0.05$; time: $t(26)=-0.50$, $p>0.05$), rearing (frequency: $t(26)=0.22$, $p>0.05$; time: $t(26)=-0.06$, $p>0.05$), head dipping (frequency: $t(26)=-0.41$, $p>0.05$; time: $t(26)=-0.36$, $p>0.05$) and grooming (frequency: $t(26)=0.17$, $p>0.05$; time: $t(26)=0.31$, $p>0.05$) (Fig. S1C, D in this document of Supplementary Information).

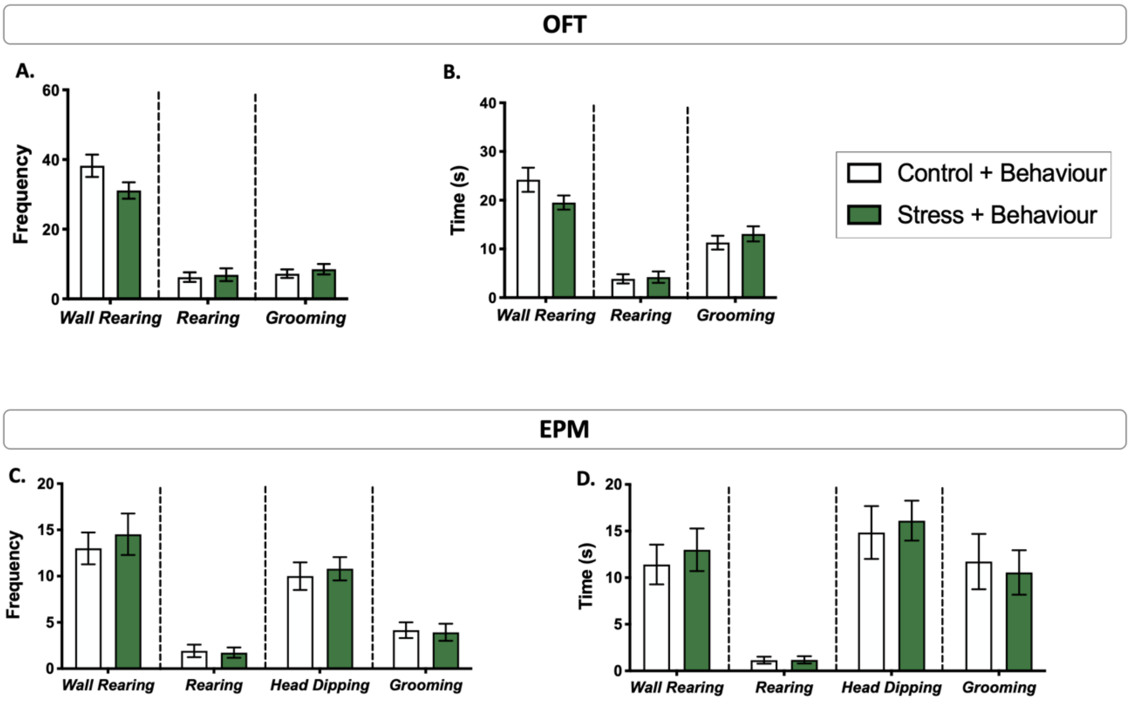

Figure S1. Complementary behavioural results for the OFT and EPM tests. This figure shows the frequency and time of ethological behaviours in the OFT (**A** and **B**, respectively) and EPM (**C** and **D**, respectively).

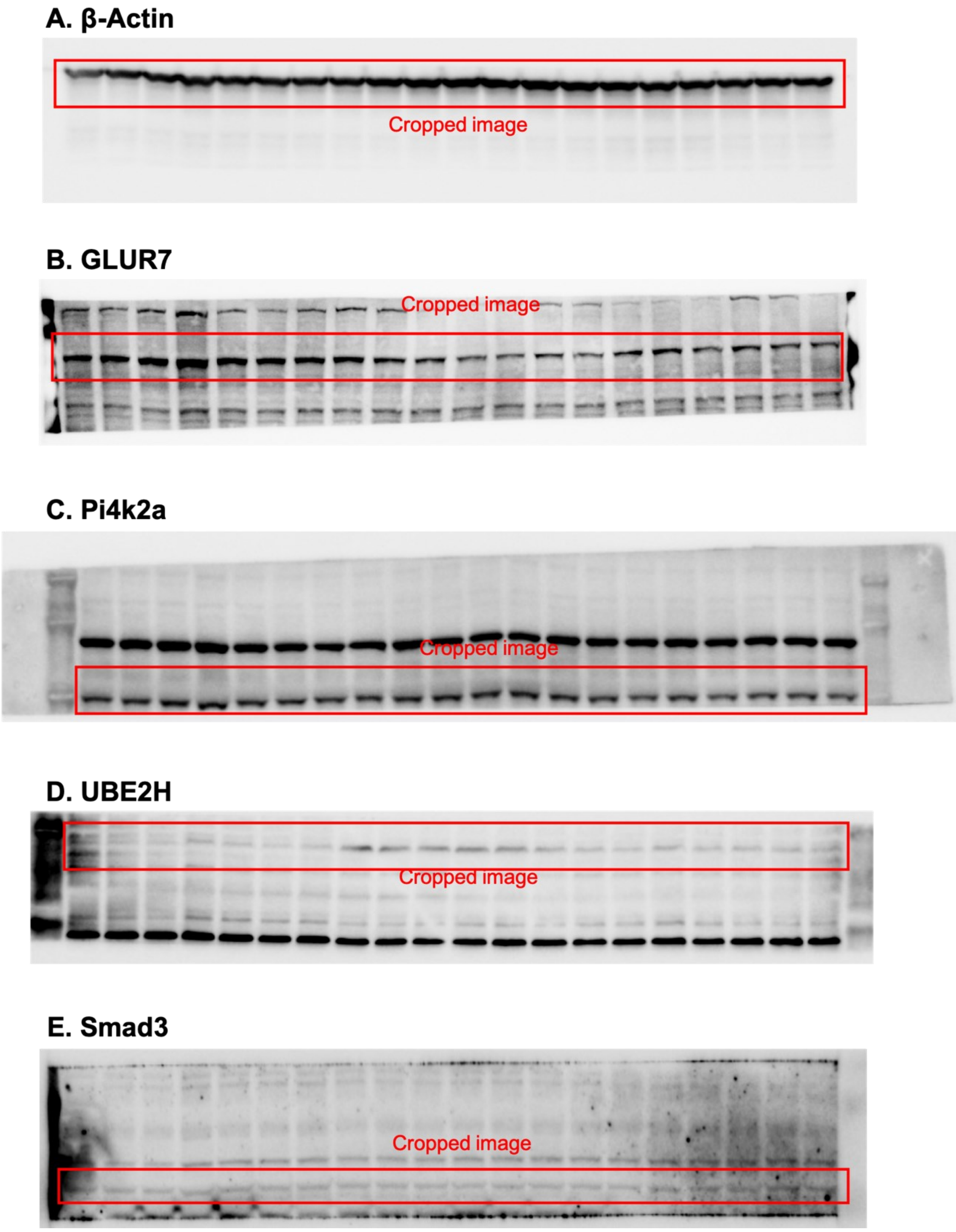

Figure S2. Western blot membranes on which the protein labelling has been cropped: β-actin (**A**), GLUR7 (**B**), Pi4k2a (**C**), UBE2H (**D**), Smad3 (**E**).
